# Evaluation of Glutathione (GSH) System in Porcine Saliva: Validation and Application of Colorimetric Method

**DOI:** 10.3390/antiox13101231

**Published:** 2024-10-14

**Authors:** Gamze Gok, Salim Neselioglu, Heng-Lun Ko, Ozcan Erel, María José López-Martínez, Xavier Manteca, Camila Peres Rubio

**Affiliations:** 1 Department of Biochemistry, Ankara Bilkent City Hospital, Ankara 06800, Turkey; gamze_gok@outlook.com (G.G.); salim.neselioglu@aybu.edu.tr (S.N.); erelozcan@aybu.edu.tr (O.E.); 2 Department of Biochemistry, Faculty of Medicine, Ankara Yıldırım Beyazıt University, Ankara 06800, Turkey; 3 Department of Animal and Food Science, School of Veterinary Science, Universitat Autonoma de Barcelona, Cerdanyola del Valles, 08193 Barcelona, Spain; henglun.ko@uab.cat (H.-L.K.); xavier.manteca@uab.cat (X.M.); 4 Department of Animal Surgery and Medicine, University of Murcia, 30100 Murcia, Spain; mariajose.lopez28@um.es

**Keywords:** antioxidants, biomarkers, glutathione system, saliva, validation

## Abstract

(1) Reduced glutathione (GSH) is considered the first line of antioxidant defense. During oxidative stress, it is oxidized to glutathione disulphide (GSSG). (2) A simple and quick spectrophotometric method based on sodium borohydride (NaBH_4_) as a reductant to measure the total and reduced GSH in porcine saliva was analytically validated and evaluated in two situations in this species: (a) in a physiological situation, involving sows during the late lactation and post-weaning periods, and (b) in a situation of sepsis in pigs experimentally induced by LPS administration. (3) The results of the analytical validation showed that the assay was precise and accurate in the porcine saliva samples. Higher total GSH and GSSG and lower reduced GSH were observed in the saliva of sows during the post-weaning period, as well as in pigs with experimentally induced sepsis. (4) In conclusion, the validated assay showed adequate analytical results and could be used to evaluate the GSH system of porcine saliva, as demonstrated during the clinical performance.

## 1. Introduction

Glutathione (GSH) is part of one of the most important cellular antioxidant responses in life forms [1,2]. The GSH system acts as the major redox buffer in most cells, being considered a key to the maintenance of aerobic life [1,3]. It participates in the maintenance of the reduced and functional forms of ascorbic acid and alpha-tocopherol during nonenzymatic reactions and in the protection against reactive species, including peroxides. This compound, as the predominant intracellular nonprotein sulfhydryl (SH) in a wide range of cells, provides protection for the SH groups of proteins from oxidation [4].

The GSH in body fluids is known as total GSH and exists in dynamically interchanging reduced and oxidized forms. Under normal steady-state conditions, the majority of GSH exists in the reduced form. The product of the oxidation of GSH by glutathione peroxidase is glutathione disulphide (GSSG), which can be considered as the oxidized form of GSH. Through the action of glutathione reductase (GSH red), GSSG can be converted back to GSH in a reaction that requires NADPH as a reductant. Overall, the total GSH includes both the reduced and oxidized (GSSG) forms [5,6].

Both a decrease in reduced GSH and an increase in GSH oxidation (GSSG) may reflect a redox unbalance, which is associated with a variety of human diseases [7,8]. The ratio of reduced GSH:GSSG is commonly used as an indicator of the redox state and, in human clinical studies, has been measured mostly in whole blood or in isolated red blood cells (RBCs) [9,10].

Saliva has been used as an alternative biofluid to measure the biomarkers of oxidative stress, including reduced GSH and GSSG, which have previously been determined in human and mice saliva samples [11,12,13]. Saliva samples are easy to obtain and are collected in a non-invasive way [14]. In addition, saliva sampling, mainly from pigs’ samples, offers the advantage of a stress-free collection process and is particularly suitable for point-of-care analysis [15,16]. In pigs, to the best of the authors’ knowledge, the evaluation of the GSH redox system has not been conducted using saliva. Instead, decreased reduced GSH has been described in red blood cells in pigs suffering from clinical and subclinical sarcoptic mange [17] and an increased reduced GSH in synovial fluid from pigs with chronic pancreatitis [18]. In heat-stressed pigs or piglets during weaning, an increased GSSG:GSH ratio was evidenced in intestinal tissues [19] and in different tissues such as the kidney, lung, liver, and distal jejunum [20,21]. In finishing pigs, the plasma GSH:GSSG ratio increased after a dietary antioxidant supplementation [22].

The measurement of reduced GSH and GSSG in biological samples is usually performed by the spectrophotometric enzymatic assay developed by Tietze [23]. However, many other assays that are more complex and specific are also employed, such as chromatographic and spectrometry methods, chemiluminescence, electrochemical techniques, and spectrofluorimetric methods [13]. The objectives of this study were (a) to perform an analytical validation of a simple and rapid spectrophotometric method using sodium borohydride (NaBH_4_) as a reductant [24] to assess the total and reduced GSH in porcine saliva, and subsequently GSSG through the half-difference between them; and (b) to evaluate potential changes in the reduced GSH, total GSH, and GSSG under two different conditions in pigs: (1) sows during the late lactation and post-weaning periods and (2) pigs with sepsis induced by LPS administration. For comparative purposes and to gain a more comprehensive understanding of changes in the glutathione metabolism in these situations, the determination of GSH reductase (GSH red) was also performed.

## 2. Materials and Methods

### 2.1. Chemicals

5,5′-dithiobis(2-nitrobenzoic acid) (DTNB), ethylenedinitrilotetraacetic acid (EDTA), hydrochloric acid (HCl), reduced L-glutathione, oxidized L-glutathione (GSSG), DTT, hydrogen peroxide solution (H_2_O_2_), 2-mercaptoethanol, methanol, NaBH_4_, sodium chloride (NaCl), sodium hydroxide (NaOH), Trizma base, and trichloroacetic acid (TCA) were obtained from Sigma-Aldrich (St. Louis, MO, USA). Ultrapure water was obtained using a purification system (Synergy water purification system, Darmstadt, Germany).

### 2.2. GSH Measurement: Total, Reduced, and Oxidized

Firstly, the proteins of the sample were removed. For that, samples were diluted (4:1) in a TCA solution (20%) to precipitate the protein content. After sample centrifugation (3000 rpm for 10 min), the supernatant was collected and stored at −80 °C until analysis.

The technique used for the measurement of GSH in the supernatant was based on the Ellman method modified by Hu [25] and was performed in two parts: (1) before and (2) after the reduction of the GSSG in the sample by using NaBH_4_ [24].

Part 1: The supernatant obtained was measured in an automatic biochemistry analyzer (Olympus AU400, Olympus Europe GmbH, Hamburg, Germany) using the Ellman method modified by Hu [25] to obtain the total GSH (reduced GSH + GSSG).

Part 2: This step consisted of performing the reduction of the GSSG content in the sample and then proceeding to the GSH measurement as indicated in “Part 1”, which gave the reduced GSH content. For the reduction, the supernatant was mixed with the reductant reagent (3.5 M NaBH_4_ and 1.5 M NaOH), and, after 15 min of incubation, 12 M HCl was added to stop the NaBH_4_ reactions. After that, the GSH measurement was performed.

The results were corrected by the dilution factors resulting from the dilution with TCA and with the reductant and stopping reagents. After that, the GSSG amount was obtained by the following formula:

GSSG = (Total GSH − reduced GSH)/2

### 2.3. Glutathione Reductase (GSH Red) Measurement

The GSH red activity was measured using a commercial kit (Randox Laboratories, Crumlin, UK). This assay is based on the oxidation of NADPH. The GSH red present in the sample catalyzes GSSG in the presence of NADPH, which is oxidized to NADP^+^. The decrease in absorbance at 340 nm is measured, and the results are expressed as U/I.

### 2.4. Saliva Collection

For the analytical and clinical performances, saliva samples were taken by using polypropylene sponges (Esponja Marina, La Griega E. Koronis, Madrid, Spain) attached to a thin, flexible metal rod, and chewed for 1 min. Subsequently, the sponges were inserted into Salivette tubes (Salivette, Sarstedt, Aktiengesellschaft and Co., Nümbrecht, Germany) and refrigerated. Upon arrival at the laboratory, the tubes were centrifuged at 3000 rpm for 10 min at 4 °C. The supernatant was transferred into Eppendorf tubes (Eppendorf Ibérica, Madrid, Spain), which were kept at −80 °C until analysis.

### 2.5. Analytical Performance

For the analytical validation of the GSH measurement, imprecision, accuracy, and sensitivity were evaluated according to previously reported protocols [26,27,28].

Imprecision: To measure the technique’s imprecision, saliva samples from 10 pigs were collected as described in “saliva collection”. The GSH system was then measured in these samples, and the three of them that presented different concentrations were selected to determine the intra- and inter-assay coefficients of variation (CVs) of the technique. The intra-assay CV was obtained from five determinations of the three selected samples (A, B, and C) in the same run. The inter-assay CV was determined from five determinations of the three samples on five consecutive days. Aliquots of the samples were freshly thawed each day to avoid freeze–thaw cycles. Coefficients of variation (CV = standard deviation (SD)/mean × 100) for the total and reduced GSH were calculated to determine imprecision.

Accuracy: The assessment of accuracy was performed by the dilution of two porcine saliva samples spiked with the matrix of interest: in this case, one with GSH and the other with GSSG. For this, a stock solution of 20 mM of GSH and GSSH was prepared in ultrapure water. Then, 18 µL of each solution was spiked into 182 µL of two different porcine saliva samples collected as described above. The spiked samples were serially diluted in ultrapure water (1:2, 1:4, 1:8, and 1:16) and then analyzed. The results were compared with those expected by linear regression analysis.

Sensitivity: The lower limit of quantification (LLOQ) for GSH and GSSG was calculated based on the lowest concentrations that could be measured with a precision of 20% and accuracy. For this, 1 mmol/L of GSH and 1 mmol/L of GSSG were serially diluted with ultrapure water until reaching a concentration of 0.003 mmol/L, and each dilution was analyzed five times within the same run.

The inter- and intra-assay CVs, as well as the accuracies of the spiked samples, should be within 15% except at the LLOQ, where a value of 20% is accepted.

### 2.6. Applicative Studies

#### 2.6.1. Sows during the Late Lactation and Post-Weaning Periods

Saliva samples from eight sows were taken between 09:00 and 10:00 in July 2019. They were taken at four different sampling points during the late lactation and post-weaning periods: on the 12th (L12) and 23rd (L23; last day of lactation) days of lactation, and on the 1st (W1) and 2nd (W2) day post-weaning. Of these eight sows, four sows were housed in a conventional farrowing system (i.e., crated during the whole lactation period), and the other four sows were housed in a temporary crating system (i.e., JLF15, produced by SKIOLD A/S, Ikast, Denmark; crated for 5 days around farrowing). The sows were in a farrow-to-finish commercial farm in the northeast of Spain.

#### 2.6.2. Experimental Sepsis

To evaluate the possible changes in the salivary GSH system of pigs with experimentally induced sepsis, samples stored from a previous study were used [29]. Briefly, these samples were from 10 growing male pigs. The animals were from the Experimental Farm of the University of Murcia (Murcia, Spain) and were in the mid-fattening period. The standard temperature of their space was 24 ± 2 °C, with a minimum space of 0.65 m^2^ per animal (Council Directive 2001/88/CE of 23 October 2001 amending Directive 91/630/CEE concerning minimum standards for the protection of pigs). They were fed a balanced diet and given water ad libitum. During the experimental period, the pigs were 14 weeks old and had a median weight of 52.45 kg (range of 46.5–61.5 kg). The control group of pigs (*n* = 5) received 2 mL of saline, and the group of pigs experimentally induced with sepsis (*n* = 5) received a single dose of 30 ug/kg LPS from *Escherichia coli* (LPS; O55:B5, Sigma-Aldrich) reconstituted in sterile saline solution; both groups received injections intramuscularly between 8 and 9 a.m.

For this study, the saliva samples obtained 24 h before the injections and at 24 and 48 h after were used.

### 2.7. Statistical Analysis

Data analyses were performed using a spreadsheet (Excel Version 10, Microsoft Corporation, Redmond, Washington, DC, USA) and GraphPad Prism (GraphPad Prism, version 8 for Windows, Graph Pad Software Inc., San Diego, CA, USA) software. Statistically significant differences in the LPS study were determined with a repeated measures one-way analysis of variance (ANOVA), followed by the uncorrected Fisher’s LSD test. Data from the pre- and post-weaning sows were analyzed by ANOVA, followed by Dunn’s multiple comparisons test. *p*-values < 0.05 were considered to be statistically significant.

## 3. Results

### 3.1. Analytical Performance

**Figure 1:**
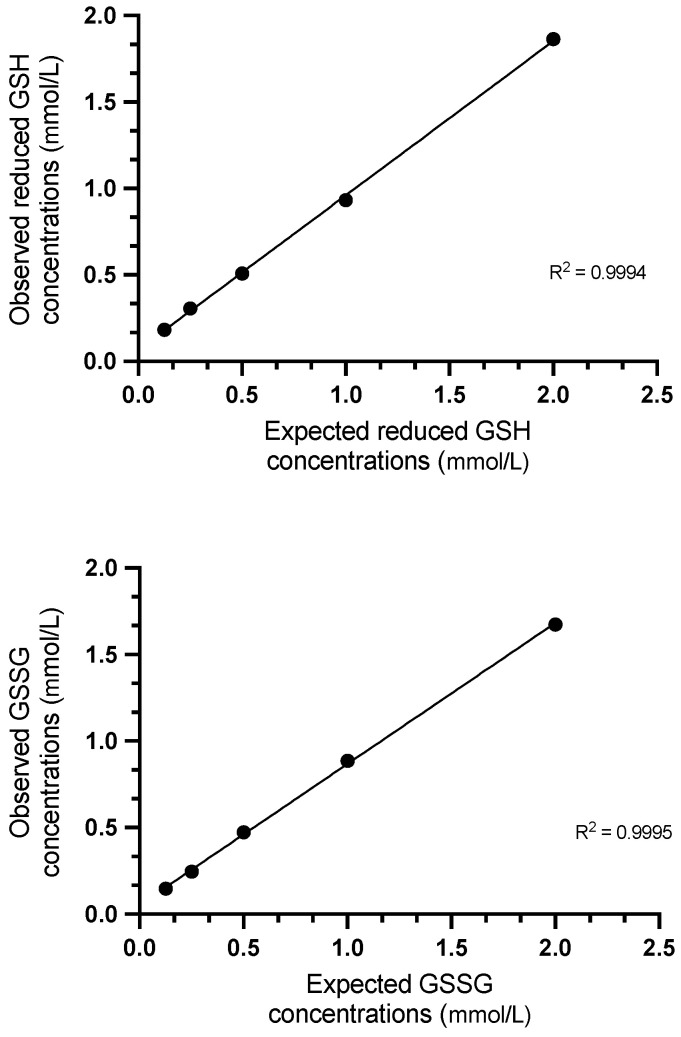
Regression line showing the reduced glutathione (GSH) and glutathione disulphide (GSSG) concentrations in pig saliva samples spiked with reduced GSH and GSSG solutions, respectively. The coefficient of determination (R2) is shown.

The intra- and inter-assay imprecision results of the total and reduced GSH and GSSG are presented in Table 1. The highest intra- and inter-assay CVs found were 5.2% and 15%, respectively. The method showed high linearity when spiked samples were diluted with ultrapure water (Figure 1). The lowest accurate and precisely measurable concentration of reduced GSH and GSSG was 0.03 mmol/L (Table 2).

### 3.2. Applicative Studies

#### 3.2.1. Sows during the Late Lactation and Post-Weaning Periods

**Figure 2:**
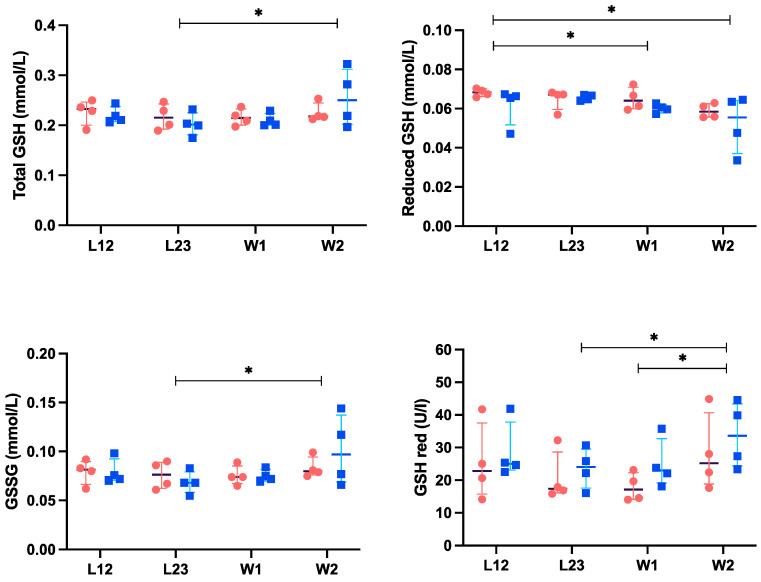
Total glutathione (GSH), reduced GSH, oxidized GSH (GSSG), and GSH reductase (GSH red) in saliva of sows during late lactation and post-weaning periods. Sows in conventional farrowing system are represented by blue squares, and sows housed in temporary crating system are represented by orange circles. L12, 12th day of lactation; L23, 23rd or last day of lactation; W1, 1st day post-weaning; W2, 2nd day post-weaning. The plots show individual values, median (black line), and 25th and 75th percentiles (colored lines) for each group of sows. * Statistically significant difference (*p* ≤ 0.05) between time points considering the data from all sows in the study.

First, the results from sows in the conventional farrowing system were compared to those from sows in the temporary crating system, and no significant differences were found (*p* > 0.05). However, when the data from all eight sows were analyzed together, a significant decrease in reduced GSH concentration was observed on days 1 (W1) (*p* = 0.03) and 2 (W2) (*p* = 0.01) post-weaning compared to day 12 of lactation (L12) (Figure 2). Total GSH, GSSG, and GSH red increased on day 2 post-weaning compared with those of the last day (L23) of lactation (*p* ≤ 0.033). In addition, GSH red showed increased concentrations on day 2 post-weaning compared with day 1 post-weaning (*p* ≤ 0.05).

#### 3.2.2. Experimental Sepsis by LPS Administration

**Figure 3:**
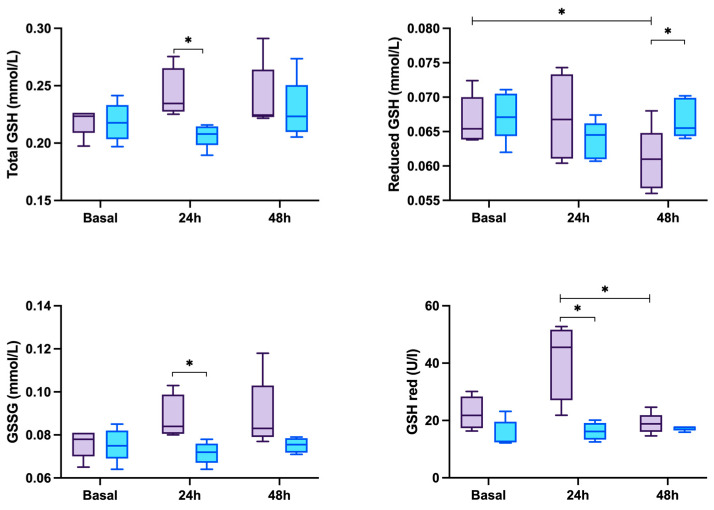
Total glutathione (GSH), reduced GSH, oxidized GSH (GSSG) and GSH reductase (GSH red) in the saliva of 5 control pigs (blue box) and 5 pigs with endotoxin lipopolysaccharide (LPS)-induced experimental sepsis (purple box). * *p* ≤ 0.05.

The total GSH, GSSG and GSH red values were higher (*p* ≤ 0.05) in the saliva of the sepsis group in comparison with the control group 24 h after the LPS administration (Figure 3). After 48 h of sepsis induction, reduced GSH was decreased in the sepsis group when compared with that at the basal time and in the control group (*p* < 0.05).

## 4. Discussion

This report describes a simple and rapid spectrophotometric method for evaluating the GSH system in the saliva of pigs. This system is related to many important cell functions, so its disturbance can affect the transcription of detoxification enzymes, cell proliferation, and apoptosis [30], which may contribute to tissue and organ dysfunction.

Since the first described method to measure sulfhydryl groups [31], many techniques have been developed to measure reduced GSH and GSSG. Techniques such as chromatography and spectrometry require sophisticated devices and are laborious and time-consuming, which limit the evaluation of the GSH redox system in biological samples. On the other hand, spectrophotometric methods, although they do not confer as elevated sensitivity and specificity as the above-mentioned techniques, are simpler and faster and do not require complex equipment. The validated assay in this study was simple to perform and did not require expensive or complex equipment, being performed using manual or automated spectrophotometers or ELISA microplate readers. Although 15 min is required to perform the GSSG reduction of the sample, the general time needed to carry out the assay is short, especially when using automatic analyzers, which permit high throughput in the sample analysis.

Regarding the analytical performance, the assay proved to be precise in porcine saliva samples as the intra- and inter-assay CVs were between 1.3 and 15%, which are acceptable from an analytical point of view [28]. The assay was linear when samples spiked with 1.8 mM of reduced GSH or GSSG were serially diluted with ultrapure water. The coefficients of determination (R2s) obtained were similar to those found for human erythrocytes or whole blood [24].

Ideally, the results obtained in this study should be compared with mass spectrometry or HPLC, which are considered gold standards; however, our assay showed robustness when analyzing the samples spiked with pure reduced GSH and GSSG solutions. In addition, a previous report showed that similar results were found when tissue samples from guinea pigs were measured for GSH and GSSG by using three different techniques: HPLC, enzymatic assays, and end-point colorimetric assays [32].

Changes in the GSH system were observed when the validated assay was applied to the saliva of sows during the late lactation and post-weaning periods. The increased total GSH, GSSG, and GSH reductase and decreased reduced GSH found on the second day post-weaning could reflect the oxidative stress reported in this physiological period. During lactation, the increased metabolic demands, physiological changes, and milk production [33,34,35] can lead to a depletion of GSH in sows [36]. Our results could indicate a potential situation of disease in sows during the lactation and weaning periods, as alterations in the GSH system are important contributors to a number of diseases [12,30]. In addition, they underscore the importance of monitoring and managing oxidative stress in lactating animals to ensure their health and productivity. Enhanced nutritional strategies, such as antioxidant supplementation, might be beneficial in alleviating oxidative stress and supporting the antioxidant defense system during lactation.

Alterations in the GSH system were also seen when evaluating the saliva of pigs experimentally induced with sepsis. It is known that sepsis is accompanied by oxidative stress and altered GSH metabolism [37]. In our study, the higher total GSH, GSSG, and GSH red, as well as the lower reduced GSH in the saliva of the sepsis group, could indicate a state of oxidative stress associated with sepsis. Similar results were found in the whole blood of children with sepsis [38] and of rats, dogs, and piglets under shock syndrome [39]. The reduced GSH is the first line of antioxidant defense against oxidative processes [40]. Our results show that this defense is depleted as a result of sepsis induced by LPS in pigs and, consequently, the GSH system is disrupted.

It should be noted that this study is preliminary. Further data regarding the validity and utility of the spectrophotometric method using NaBH_4_ for evaluating GSH and GSSG in porcine saliva should be collected, and a comparison with other established gold standard techniques should be conducted. Furthermore, it would be valuable to conduct additional studies to evaluate the GSH system in the saliva of pigs affected by other diseases and under various physiological conditions.

## 5. Conclusions

Our results show that the GSH system can be evaluated precisely and accurately in porcine saliva through the spectrophotometric method using NaBH_4_ as a reductant. In addition, two different conditions in sows and pigs, such as the late lactation and post-weaning periods, and experimentally induced sepsis produced increases in the GSSG:GSH ratio, which reflects oxidative stress and was detected by the validated assay.

## Figures and Tables

**Table 1:** Mean, standard deviation (SD), and intra- and inter-assay coefficients of variation (CVs) of reduced glutathione (GSH), total GSH, and oxidized GSH (GSSG) in three different porcine saliva samples (A, B, and C).

Biomarker	Saliva Sample	Intra-Assay Imprecision			Inter-Assay Imprecision		
		Mean (mmol/L)	SD	CV (%)	Mean (mmol/L)	SD	CV (%)
Reduced GSH	A	0.039	0.001	2.7	0.032	0.004	15
	B	0.046	0.002	5.2	0.041	0.004	9.8
	C	0.065	0.002	2.8	0.062	0.005	8.0
Total GSH	A	0.209	0.005	2.4	0.209	0.006	3.3
	B	0.223	0.003	1.3	0.225	0.005	2.3
	C	0.263	0.005	2.1	0.257	0.006	2.6
GSSG	A	0.107	0.003	2.8	0.109	0.002	2.2
	B	0.085	0.002	3.2	0.088	0.004	4.7
	C	0.071	0.002	2.5	0.071	0.002	2.3

**Table 2:** Reduced glutathione (GSH) and oxidized glutathione (GSSG) concentrations, standard deviations (SDs), and coefficients of variation (CVs) obtained in the sensitivity experiment.

Reduced GSH (mmol/L)				GSSG (mmol/L)			
Expected	Obtained (Mean; *n* = 5)	SD	CV (%)	Expected	Obtained (Mean; *n* = 5)	SD	CV (%)
1.0000	0.974	0.008	0.774	1.0000	1.093	0.081	7.451
0.5000	0.484	0.001	0.271	0.5000	0.573	0.051	8.971
0.2500	0.243	0.003	1.175	0.2500	0.326	0.016	5.047
0.1250	0.120	0.002	1.577	0.1250	0.164	0.012	7.455
0.0625	0.059	0.002	2.844	0.0625	0.073	0.006	8.025
0.0313	0.029	0.001	3.684	0.0313	0.028	0.004	12.437
0.0156	0.013	0.003	22.777	0.0156	0.013	0.003	23.435
0.0078	0.006	0.002	26.727	0.0078	0.009	0.008	93.635
0.0039	0.002	0.002	66.946	0.0039	0.001	0.002	121.112

## Data Availability

The raw data supporting the conclusions of this article will be made available by the authors on request.
